# Congenital Word-Blindness

**Published:** 1934

**Authors:** E. R. Chambers

**Affiliations:** Ophthalmic Surgeon, Bristol Royal Infirmary; Surgeon, Bristol Eye Hospital


### CONGENITAL WORD-BLINDNESS.

BY

E. R. Chambers, F.R.C.S.,

*Ophthalmic Surgeon, Bristol Royal Infirmary; Surgeon, Bristol Eye Hospital.*

---

The importance of this condition lies in the fact that the cases are by no means uncommon, and that if the condition is recognized the child is saved much misery and the parents spared the expense and trouble of endeavouring to educate a child who through no fault of his own is incapable of achieving the results that are expected of him. The child will often develop an inferiority complex, as he cannot keep pace with children of his own age, his classmates holding him up to ridicule and his teachers regarding him either as a fool or as one who will not take the trouble to learn. I have had a teacher confidentially explain to me that it is quite obvious that the child is not taking pains, and that there is no reason why a healthy, robust child, who is intelligent in everything but reading, makes such a bad performance. On the other hand, the child will say that he dreads reading lessons, particularly if he is asked to read aloud The following are two instances of this type of case :—

A healthy and intelligent-looking boy, aged 11 years, was brought to me with a history that, although he had been at school for four years, his reading was still atrocious, and his form-master felt that there must be something wrong with his eyes to account for the trouble. He was very good at figures, and got on well with arithmetic ; as a hobby he was very fond of mechanics, and he had an excellent memory. There was nothing of note in his family history, his parents had never had any trouble with reading, and his sister, the only other child, was quite normal. On questioning, the boy stated that he knew all his letters perfectly, but when they were put into words, particularly if these happened to be long, he seemed to have forgotten what the word was or what it meant. When he was asked to read aloud in class he had to go very slowly, and was able to get the meaning of the words much better if he was given time to spell them to himself. When he was reading to himself he found it much easier to spell the words aloud. He did not like books, and would never think of reading for pleasure, but he liked being read to, and had no difficulty in memorizing the contents of a book if he had not to read it himself. He could recognize quite normally objects, persons and places. Pictures of all kinds he recognized at once. On being given a simple reading test he appeared to take it as a foregone conclusion that he had encountered his old difficulty. He put the book first close to his eyes, then pushed it away and shifted it in different positions, as if he were trying to get a better light. On being questioned, he assured me that he could see even the smallest print with ease, and in order to make sure of this I asked him to spell one of the longer words ; this he did with ease. On commencing to read he had no difficulty with the short, familiar words, but with unfamiliar words, even if they were short, he would hesitate, while with longer words he had to stop and spell them to himself. His vision was normal. There was no error of refraction, nor was there any abnormality in the muscle balance.

Another case occurred in a girl aged 10, who had been three years at school ; she was robust and healthy in every way. Her mother stated that her birth was normal, and that she had had measles, chicken-pox and whooping-cough. There had been the greatest difficulty in teaching her the alphabet, and at the end of her first year's schooling she had not learnt it properly. She seemed to have learnt it several times, but had forgotten it, and even now she would sometimes forget what a letter was, particularly if it was part of a longish word. When I saw her she knew all her letters properly, and had no difficulty with the short, familiar words, but with longer ones and even short and rather unfamiliar words she would spell them out slowly to herself, and took some time to make up her mind what they spelt. In her education the only difficulty that had been encountered had been this trouble with reading. Her general intelligence and memory were good. She was good at all handwork and sewing, and had no trouble with figures. She told me that she could see perfectly even the smallest print, and knew all the letters of a word perfectly, but that when the letters were put together in a word she could not remember what the word meant. Sometimes she would be better than at others, and on her bad days she found reading very difficult. She wanted to read, and had done her best to overcome her difficulty, but she disliked reading because she found it such hard work. If she could she always tried to get someone to read to her. Her eyes were sound and healthy, the refraction showing a small amount of hypermetropia which I corrected with reading glasses. There was no error in the muscle balance. I advised that this child should be removed from school and treated privately. She made excellent progress, and is now able to read fluently, but she tells me that even now when writing she sometimes gets the letters of a long word wrong because the sound of a word does not convey to her the necessity of forming it with its correct letters. She has frequent recourse to a dictionary for unfamiliar words.

It has been estimated by Thomas that one in a thousand children in the elementary schools suffer from this condition, and Fisher states that of thirty-three reported cases of congenital word-blindness twenty-four occurred in boys and nine in girls. In some cases heredity would appear to play a part.

Hinshelwood, in discussing the possible cause of this condition, states that the difficulty in learning to read is due to some congenital defect in the visual memory centre for words and letters in the left angular and supra-marginal gyri. In learning to read by sight there are two distinct stages. In the first stage individual letters of the alphabet are stored up. The memory for words is first registered in our auditory memory, that is, we are able to spell a word before we are able to recognize the word by sight alone.

When the individual has acquired in his visual memory a knowledge of the individual letters he is able to read words by spelling out aloud each letter and thus, by appealing to his auditory memory, he gets the proper word. The second stage in reading consists in the gradual acquirement and storage of the visual memory for words; when this is accomplished the individual reads by recognizing each word as a separate picture. In the cases under discussion the difficulty in reading arises owing to the fact that their visual memory for words and letters is congenitally deficient, the degree of difficulty depending on the state of development of the visual memory centre.

Fisher, on the other hand, suggests that all these cases are not congenital in origin, but may be due to birth injuries; that a history of difficulty at birth should be inquired into, and whether there is any history of paralysis, convulsions or fits.

In the diagnosis of these cases it is important to remember that the parents or school authorities assume that there must be something wrong with the child's sight to account for the difficulty in learning to read. When these children are being examined they must not be allowed to spell out the word letter by letter nor to move the lips silently, as by adopting these methods they assist their defective visual memory by appealing to their auditory sense. In a bad case the child may not be able to recognize a simple word of three letters, but he knows the individual letters and how to spell the word, and if he is given time to spell the word either aloud or by moving his lips he can say what it is.

As regards treatment, these children cannot be taught in a class with others. They have probably been at school some time before the defect is discovered, and owing to their slowness may have been held up to ridicule by the other children and regarded by their teachers as those who will not take the trouble to learn, so that many of them have been made to feel that they are inferior to others of their own age. They must be taught alone, and it is surprising the progress that will be made if this is done by a teacher who is in sympathy with the child. The reading lessons should not be carried out for any length of time at one sitting, but a method should be adopted of short periods of work with intervals in between. There is no doubt that attempts at reading take a lot out of the child, and he rapidly fatigues. In the more severe cases large block letters should be used at first, as these are unquestionably more easily impressed on the child's memory. It will be readily understood that much depends on the type of person who is chosen to teach the child ; she should, if possible, be interviewed by the doctor in charge of the case and all the circumstances should be carefully laid before her.

If these cases are recognized reasonably early, and adequate and considerate treatment is carried out, there is no reason why at a later date they should not return to school and enjoy the education of a normal individual. The time taken for these children to read reasonably well varies enormously, and many, after their education has been successfully completed, perhaps never read with the pleasure of a normal individual, while many others have all their lives more difficulties with spelling than is usual. It should be realized that all grades of this condition exist ; the severe and obvious types are easily detected, but the milder cases are readily missed, and the possibility of their existence should always be considered in examining a child who has difficulty in reading and whose refractive error is not sufficient to account for his troubles.

BIBLIOGRAPHY.

Hinshelwood, J., *Congenital Word-Blindness*, London, 1917.

Nettlewhip, E., *Ophthalmic Review*, 1901, xx. 61.

Hinshelwood, J., *Ophthalmic Review*, 1902, xxi. 91.

Hinshelwood, J., *Lancet*, 1900, i. 1,506.

Hinshelwood, J., *Brit. Med. Jour.*, 1904, ii. 1,303.

Fisher, J. H., *Trans. Ophthalmic Society, United Kingdom*, 1909–10, xxx. 216.

Thomas, C. J., *Public Health*, 1908, xxi. 96.

Fildes, L. G., *Brain*, 1921, xliv. 286.

Whipham, T. R., *Ophthalmoscope*, 1916, xiv. 11.

